# A Narrative Inquiry into Junior Nurses’ Psychological Recovery After Adverse Events in China

**DOI:** 10.3390/healthcare14060707

**Published:** 2026-03-10

**Authors:** Lu Qi, Jiaxi Xu, Aimei Mao

**Affiliations:** 1 Ordos Central Hospital, No. 23 Yijinhuoluo West Street, Dongsheng District, Ordos City 017000, China; 2 Kiang Wu Nursing College of Macau, Avenida do Hospital das Ilhas No. 447, Coloane, RAEM, Macau 999078, China; phd2025006@stud.kwnc.edu.mo

**Keywords:** nurses, adverse events, second victim, psychological recovery, qualitative research

## Abstract

**Highlights:**

**What are the main findings?**

- Junior nurses experience their most intense distress immediately after an adverse event, underscoring the importance of timely emotional support.
- Nurse managers are well-positioned to create supportive environments where early-career nurses feel valued and understood.

**What are the implications of the main findings?**

- Although the concept of a “no-blame” culture is increasingly recognized within China’s healthcare system, a fully realized just culture that balances accountability and fairness remains limited.
- Building a genuine, just culture requires visible leadership commitment, targeted education, and transparent communication.

**Abstract:**

**Background**: Junior nurses—who are most prone to errors and emotional distress as “second victims”—often experience underreported adverse events and psychological challenges. This study aimed to explore how junior nurses in China psychologically recover after adverse events and how they navigate their recovery experiences. **Methods**: Guided by Scott’s Second Victim Recovery Trajectory Model, a qualitative study was conducted from September to November 2023. Purposive sampling was used to recruit junior nurses from hospitals across China who had experienced adverse events in their clinical practice. Informed by themes emerging from the initial nurse interviews, the study subsequently included nurse managers to provide additional organizational and managerial perspectives. In-depth semi-structured interviews were conducted with nine junior nurses and four managers. Data were analyzed thematically within a narrative inquiry framework and reported following the Standards for Reporting Qualitative Research (SRQR). **Results**: Two main themes emerged: (1) the trajectory of adverse events: A nonlinear psychological response, and (2) final outcomes of adverse events: reflection and adjustment. Psychological recovery broadly followed the Second Victim Recovery Trajectory, with only two outcomes observed—dropout and thriving. Organizational support sometimes enabled nurses to bypass certain recovery stages, but such support was limited. **Conclusions**: Junior nurses experience notable emotional distress following adverse events. The post-event recovery is strengthened by supportive interpersonal environments but limited by insufficient organizational attention. The study findings highlight the need to foster a just, blame-free culture to promote recovery.

## 1. Introduction

Adverse events (AEs) not only harm patients but can also traumatize the healthcare providers involved. Wu termed such affected providers “second victims,” highlighting that clinicians often experience guilt, fear, self-doubt, and symptoms of post-traumatic stress after an error [1]. Subsequent research has confirmed that these emotional injuries can harm a provider’s well-being and performance, leading to burnout or leaving the profession if unaddressed [2,3,4].

Less experienced nurses are more prone to medical errors and less likely to report them than senior nurses [5]. This vulnerability is often attributed to limited clinical experience and insufficient exposure to recurring patient care patterns, particularly during the first three years of practice [6]. In mainland China, junior nurses account for most AEs, with approximately 75–86.7% occurring in this group and nearly 80% within their first three years of employment [7,8].

The management of AEs in China follows a structured, hierarchical process guided by national regulations and institutional protocols (Chinese Hospital Association, 2018; National Health Commission of the PRC, 2024) [9]. Based on these directives, healthcare institutions implement localized measures. Upon identification of an AE, procedures generally involve reporting, investigation, documentation, and assignment of accountability (National Health Commission of the PRC, 2023) [10] (Table A1). The urgency and composition of investigation panels vary depending on event severity, with reports directed to different authorities.

The recovery process for healthcare professionals who experience AEs—referred to as “second victims”—is a complex psychological journey. Scott’s Second Victim Recovery Trajectory Model [11] outlines six stages of this recovery process: (1) Chaos and Accident Response: Victims experience initial shock, confusion, and emotional distress; (2) Intrusive Reflections: Victims are haunted by persistent thoughts, guilt, and self-doubt; (3) Restoring Personal Integrity: Victims seek reassurance and support from colleagues, family, or other sources of comfort; (4) Enduring the Inquisition: Victims suffer stressful investigations, which can intensify emotional vulnerability; (5) Obtaining Emotional First Aid: Victims actively seek support through counselling or peer support programs; and (6) Moving On: Victims leave the profession (dropout), continue with lingering emotional scars (survive), or achieve personal or professional growth (thrive) as the result of the event experience.

Despite the central importance of patient safety in healthcare systems, empirical research focusing on AEs involving junior nurses remains limited. In particular, there is a notable scarcity of qualitative studies within the Chinese healthcare context. Existing literature has largely emphasized the prevalence and reporting of errors [7,8], with comparatively little attention paid to the psychological recovery processes that follow such incidents. Furthermore, few studies have explored second victim experiences from a culturally situated perspective or explicitly examined how established frameworks, such as Scott’s Second Victim Recovery Trajectory, manifest within specific organizational and managerial environments. This gap highlights the need for context-sensitive qualitative inquiry into junior nurses’ post-event experiences.

Guided by Scott’s Second Victim Recovery Trajectory Model, this study examined the psychological recovery of junior nurses. Specifically, the study aimed to fill a gap in narrative qualitative research on junior nurses in China by exploring psychological recovery trajectories after AEs within culturally and organizationally situated contexts. Therefore, the study sought to answer the following two questions: (1) How do junior nurses in China narratively construct their psychological recovery after involvement in AEs? (2) How do these narratives align with or diverge from Scott’s model?

## 2. Methods

### 2.1. Design

This study adopted an interpretivist paradigm within a qualitative narrative research design, focusing on junior nurses’ personal experiences and psychological recovery journeys following AEs. Consistent with an experience-centered analytic orientation, adverse events were approached as psychologically significant situations as defined by participants, rather than being differentiated according to technical severity. This study was prepared following the Standards for Reporting Qualitative Research (SRQR) [12].

### 2.2. Participants

A purposive sampling of junior nurses was recruited, and personal social networks were also leveraged to facilitate recruitment. Efforts were made to ensure diversity in participants’ locations, years of work experience, and types of healthcare institutions. Before the interviews, communication with interested participants took place via the social media app WeChat, where the shared professional background between the researchers and participants helped establish trust quickly. All invitations to participate were provided on a one-to-one basis.

The inclusion criteria were (1) Chinese registered nurses, (2) those who had no more than three years of clinical nursing experience, and (3) nurses who had been involved in AEs during their practice.

### 2.3. Data Collection

In-depth semi-structured interviews were conducted between September and November 2023. The interview guide was developed through a review of relevant literature and team discussion, focusing on the nursing AEs, emotional impacts, and coping strategies (Table 1). A pilot interview was conducted with a nurse who was not a member of the research team to test the interview guide. Based on the feedback, minor adjustments were made to improve the clarity and relevance of the initial questions. The revised guide was subsequently used for all formal interviews. The results of the pilot interview were not included in the analysis, and the participant was not invited to take part in the study.

Initial interviews with five junior nurses underscored the pivotal role of nurse managers in AEs management. In response, and to enable triangulation of organizational perspectives and enhance interpretive depth, nurse managers were subsequently recruited [13]. Their interviews were conducted after those with junior nurses to further elaborate on emerging themes.

Thirteen participants who met the inclusion criteria took part in the interviews, including nine junior nurses and four nurse managers. None of them had come from the same healthcare institution. They had no direct supervisory or reporting relationships. Four of the junior nurses had a previous classmate relationship with the primary researcher. All nurse managers were familiar with members of the research team but had no professional interactions or conflicts of interest. Two nurse managers and some interview participants were from the same hospital, but they were unaware of each other when invited to participate. The primary researcher used WeChat 8.0.41 to schedule interviews. Of the thirteen participants, five opted for face-to-face interviews and eight for online video interviews. Face-to-face interviews were conducted in private seminar rooms outside participants’ work areas, with opaque windows to ensure strict confidentiality. All interviews, including online sessions conducted via a secure, password-protected platform (Tencent Meeting) or a private video-enabled social media app (WeChat), were held with only the primary researcher and the participant present; among online participants, five all declined to turn on their cameras. Interviews lasted 30–60 min and were audio-recorded on the primary researcher’s phone with participant consent. Data collection continued until sampling saturation was reached, with no new perspectives emerging during the interviews. Saturation was monitored iteratively by the research team to ensure an adequate depth and breadth of data for the research aims.

### 2.4. Data Analysis

**Figure 1:**
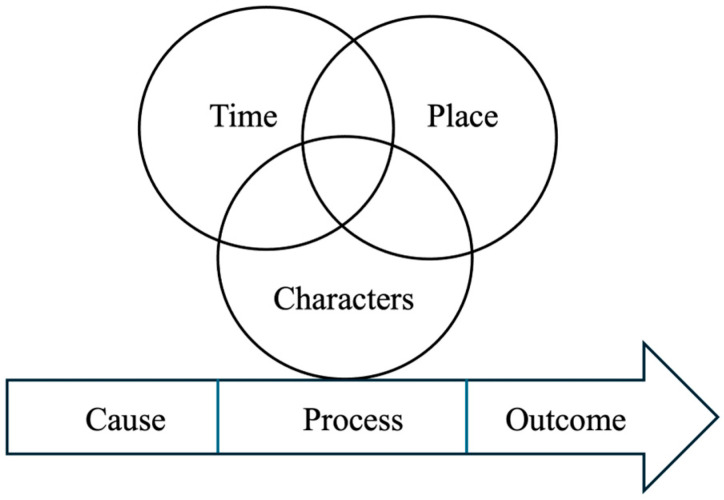
Key Elements from the Interview.

Data were analyzed within a narrative inquiry framework guided by Clandinin and Connelly’s three-dimensional narrative structure, considering temporality, sociality, and place [14]. The analytic steps were as follows: (1) Interviews were read and reread to gain a comprehensive understanding of each participant’s experiences with AEs. Events within each narrative were organized to reflect their temporal sequence (time), contextual setting (place), characters involved, and the cause–process–outcome continuity (see Figure 1). (2) Skeletal narratives were drafted to outline the chronological sequence of events for each participant (see Appendix B). (3) Across all narratives, recurring patterns and themes were identified while ensuring that each story retained its integrity and temporal, social, and situational dimensions. Themes were then cross-referenced with the skeletal narratives to enrich interpretation and maintain alignment with the narrative inquiry framework. Conceptual saturation was considered achieved when no new codes or interpretive insights emerged from subsequent interviews during iterative analysis across the skeletal narratives. NVivo 12.0 software was used to facilitate data analysis.

### 2.5. Ethical Considerations

The study’s ethical approval was approved by the Ethics Committee (Ref. #REC-2023.01), which followed the principles of the Declaration of Helsinki and subsequent modifications [15]. Before commencing the study, participants who opted to participate provided their written informed consent. They were afforded the continual option to withdraw at any moment while also ensuring the preservation of their confidentiality and anonymity.

### 2.6. Trustworthiness

To ensure the trustworthiness of the analysis, credibility, transferability, confirmability, and dependability were addressed in accordance with established qualitative criteria [13].

Credibility was enhanced through data triangulation by recruiting nurse managers to incorporate organizational perspectives, thereby reducing potential bias from reliance on a single data source. Additional strategies included field notes by the primary researcher, member checking with three participants for transcript and thematic verification (with no modifications requested), and ongoing peer debriefing with an experienced qualitative supervisor.

Transferability was supported through rich descriptions of the study context and participants, purposive sampling across provinces, inclusion of nurse managers for additional perspectives, verbatim quotations to illustrate findings, and systematic verification of reporting for completeness and accuracy.

Confirmability was ensured through ongoing reflexive self-awareness by the primary researcher, a master’s candidate trained in qualitative research methods, who occupied an insider–outsider positionality. While sharing a professional nursing background that facilitated rapport, the researcher held no hierarchical or workplace relationship with the participants. Coding decisions were discussed with co-authors who were not part of the same professional network to enhance analytic distance.

Four participants had a prior classmate relationship with the primary researcher. This prior connection may have shaped participants’ openness or constrained expressions of institutional criticism. To address this potential influence, the researcher maintained a reflexive journal documenting assumptions and emotional responses after each interview. All participants were approached and treated equally. Interview questions were posed in a neutral and non-leading manner, and interpretations were grounded in verbatim transcripts to minimize selective emphasis. Ongoing discussions within the research team further supported critical reflection on how relational dynamics might have influenced data production and interpretation.

Dependability was established through independent dual coding by two researchers who reached consensus in coding frame, regular supervision meetings to review methodological decisions and ensure procedural consistency, and maintenance of an audit trail documenting the data analysis process.

## 3. Results

### 3.1. Characteristics of Participants

One junior nurse chose not to disclose the name of the employing institution. The other 12 participants came from six provinces across China, and most of the overall sample (*n* = 13) worked in Class 3A hospitals—the country’s highest-tier facilities. Among the nine junior nurses, six had less than one year of experience, and three had about three years, underscoring their limited clinical tenure; eight of them rotated night shifts. The four nurse managers included three head nurses and one nursing director. Participant demographics are summarized in Table 2.

### 3.2. The Trajectory of Adverse Events: A Nonlinear Psychological Response

The junior nurses vividly recalled their involvement in AEs, though some were unclear about which specific incidents qualified, suggesting that some had experienced multiple of these events. While a few self-reported the events, others were informed by colleagues or patients and were subsequently required to participate in the reporting process. Regardless of patient outcomes, all junior nurses faced disciplinary actions, such as warning tickets, salary or bonus deductions, or mandatory self-reflection reports. Although these penalties were described as minor and temporary, the nurses acknowledged experiencing significant negative emotions. Nurse managers, when reflecting on similar situations, described their responses primarily in developmental terms, emphasizing learning and professional growth. We divided junior nurses’ psychological response to the “AE trajectory” into five stages and identified two outcomes. Participants’ experiences did not consistently follow a fixed or strictly sequential order. The boundaries between stages were often indistinct, with nurses describing overlapping emotional and cognitive responses.

#### 3.2.1. Stage of Chaos and Accident Response

At the onset of the AEs, participants reported experiencing a range of negative emotions, including panic, stress, and anxiety, largely attributed to uncertainty regarding potential consequences. They subsequently engaged in immediate corrective actions aimed at minimizing patient harm. For example, Nurse L recounted that when a patient under her care experienced cardiac arrest, she initially felt anxious but promptly alerted physicians and nearby colleagues, who then assisted with resuscitation efforts.

“*I urgently knocked on the door to call for the colleagues in the Intensive Care Unit ward to come help… I was extremely panicked and didn’t know what to do. It was my first time encountering a patient with such severe hemoptysis, and I had no idea how to handle the situation.*”

(Nurse L)

#### 3.2.2. Stage of Intrusive Reflections

At this stage, nurses’ experiences were not clearly bounded. Some participants reported recurrently recalling and reassessing the circumstances leading to the AEs, often accompanied by negative emotions such as regret and remorse. For instance, Nurse P described an AE that occurred when she returned from lunch. Upon arriving at the ward’s nurse station, she found that one woman was waiting to be admitted. However, due to a malfunction in the e-admission system, the patient had to wait for an extended period. The patient’s husband later filed a formal complaint with hospital administrators. Nurse P repeatedly recalled the situation and regretted returning from lunch too soon.

“*If I had known about the situation, I wouldn’t have rushed through my lunch that day.*”

(Nurse P)

However, Nurse S showed no signs of regret or remorse during the investigation into the missing sleeping pill. She attributed the medication discrepancy to the ward’s management issues rather than her personal wrongdoing:

“*Even if I knew the medication might be misplaced, it’s somewhat unavoidable. I can’t possibly stay in the medication storage room 24/7, neglecting all my other responsibilities—that’s unrealistic.*”

(Nurse S)

#### 3.2.3. Stage of Restoring Personal Integrity

Not all the nurses had experienced the same process in this stage, but most involved junior nurses frequently expressed concerns about their professional competence being questioned by superiors or colleagues. Nurse L experienced a significant event when a patient collapsed in the washroom. She became anxious about losing the trust of the nurse manager and her colleagues:

“*I felt like the head nurse thinks I am not very capable, and the senior colleagues see me as unreliable. I am worried they won’t want to work shifts with me in the future.*”

(Nurse L)

Nurse managers, when reflecting on similar situations, stated that they approached the junior nurses who made mistakes with goodwill. Head Nurse H1 dismissed the idea that the juniors would be judged based on their errors.

“*Sometimes, we may be frustrated with a junior nurse who makes a mistake due to carelessness. But time will reveal their true abilities. For the juniors, the key is learning from their mistakes to improve and grow. As they make progress, they can regain our trust.*”

(Head Nurse H1)

Notably, a few junior nurses expressed that they were not worried about losing their colleagues’ trust. Nurse F, who was involved in an incident where she forgot to suction a thrombosis, was confident that the accident would not affect her colleagues’ trust in her:

“*I’ve never been worried that my colleagues wouldn’t want to work with me after the incident. Our team has a great, relaxed dynamic…, and we even go out for team-building meals sometimes.*”

(Nurse F)

#### 3.2.4. Stage of Enduring the Investigation

At this stage, the involved nurses found the investigation period extremely unbearable, experiencing varying degrees of negative emotions. For example, Nurse L1 suffered from severe anxiety and insomnia after the incident when he administered the wrong medication. While he was concerned about the impact of the error on the patient, the rumors among his colleagues about the consequences only worsened his distress:

“*At the very beginning, some nurses told me how serious the situation could be, which really worried me. When I went home, I couldn’t sleep at all. I kept thinking about how I would explain myself if the head nurse questioned me. It was incredibly anxiety-inducing and nerve-wracking.*”

(Nurse L1)

To his relief, the wrong medication had no adverse effects on the patient, and the patient did not file any complaints. As a result, Nurse L1 received an oral warning from the head of the ward.

#### 3.2.5. Stage of Obtaining Emotional First Aid

At this stage, the involved nurses often sought emotional support and assistance from external sources. However, a few junior nurses were unwilling to share the AEs with others, as they found the revelation shameful. Some turned to their families for support but felt disappointed, as their families did not fully understand the nature of the events. Similarly, a few junior nurses were dissatisfied with the responses they received when reaching out for help from their senior peers, as Nurse W expressed:

“*They (the senior colleagues) say that mistakes are a part of every nurse’s career. Because of that, they didn’t take the time to listen to my side of the story or offer any suggestions.*”

(Nurse W)

Additionally, most junior nurses expressed a strong desire for support from their head nurses but often felt that such support was lacking. Nurse L felt doubly hurt—both by the patient and the head nurse:

“*After the patient complained, the head nurse reprimanded me again. She even criticized me in front of everyone during the morning briefing.*”

(Nurse L)

Although junior nurses wanted support from nurse managers, few approached them because they were concerned about further disclosures or criticism. However, head nurses described a more benign way to support junior nurses. Head Nurse H2, who had been a nurse leader for more than 20 years, described their system as following a non-punitive approach:

“*In most cases, we adopt a non-punitive approach. When a nurse is involved in an adverse event, I support her by adjusting the roster—giving her time to recover and temporarily assigning her away from critical shifts*.”

(Nursing Manager H2)

#### 3.2.6. Final Outcomes of Adverse Events: Reflection and Adjustment

Across the narratives, two outcome patterns—reflection and adjustment—were evident. Notably, no participant described a prolonged state of emotional endurance or suspended coping without clear withdrawal or growth-oriented reengagement. While several participants recounted distress and temporary hesitation, these accounts either evolved into reflective adjustment or culminated in withdrawal, rather than stabilizing into an intermediate “survival” position.

##### Reflection on Specific Situations

Nearly all the junior nurses reported learning valuable lessons from the AEs. For example, Nurse X accidentally turned on the ultraviolet sterilizer without ensuring all patients had left the area, resulting in a 50% deduction from her monthly bonus. Following the incident, she began researching learning materials on UV disinfection and said:

“*It (the incidence) gave me new insights—like understanding the environmental requirements for ultraviolet disinfection and how it affects personnel exposed to it. I feel much more knowledgeable now.*”

(Nurse X)

##### Reflection on Career Adjustment

In contrast, Nurse S chose to leave her current workplace and seek a better workplace fit after an AE:

“*I feel that I’m not well-suited to my current ward, so I plan to go to another one instead.*”

(Nurse S)

## 4. Discussion

This study revealed that junior nurses were primarily involved in AEs with minor or no impact on patient safety, and these are common in clinical settings. Several participants reported hesitation to report minor AEs, suggesting potential underreporting, which aligns with previous literature [16]. This reluctance may be rooted in culturally mediated factors such as face-saving and hierarchical power distance, which have been shown to influence experiences of guilt and silence and to significantly affect the relationship between safety emphasis and fear of error reporting among Chinese nurses [17,18]. The study provides a contextually grounded examination of how junior nurses in China experience and navigate psychological recovery following involvement in minor AEs. In particular, recovery trajectories appeared fluid, stage boundaries were frequently indistinct, and adaptive interpretations often coexisted with organizationally shaped emotional responses.

Participants in our study reported that the initial period following an AE was particularly stressful. Moreover, the principle of “patient-centered care” has led to heightened attention to the first victim (the patient), often prioritizing their needs above all else. This focus may have overshadowed the challenges faced by second victims. Studies have shown that adverse emotional reactions are most dramatic in the early stages of the event, with the recovery process lasting several months or even years [19].

Several respondents reported a comparatively smoother post-event recovery, crediting a supportive institutional environment—a departure from the more arduous trajectory described in Scott’s model. This observation aligns with evidence emphasizing the role of social support in facilitating recovery from AE-related distress [20]. The findings indicated that participants generally perceived managerial support as beneficial in facilitating their recovery. Nurse managers were viewed as key in addressing factors affecting second victims and providing both immediate and sustained psychological support, in line with existing literature [21,22]. However, many junior nurses reported hesitation in approaching their supervisors. This finding may reflect broader concerns associated with blame cultures and potential professional repercussions, as noted in previous studies [23]. The divergence on the concept of blame between junior nurses and nurse managers suggests that perceptions of blame or support depend more on how institutional responses are enacted and experienced than on formal policy declarations. Participants reported that peers can also serve as a supportive source during post-AE recovery, supporting the finding by Kiviliene et al. [20].

Scott et al. [11] identified three possible outcomes following AEs: dropout, survival, and thriving. However, our study observed only two outcomes: dropout and thriving. Although dropout may seem a passive copying outcome, those who chose to leave explained that their decision was motivated by a desire to find healthcare institutions or departments better aligned with their career development goals. This suggests that junior nurses’ reflection and adjustment extended beyond event-specific situations and into their long-term career development [24,25].

The absence of a clearly articulated ‘survival’ state warrants contextual interpretation. From a cultural perspective, professional identity construction among junior nurses in China may have potentially encouraged the reinterpretation of distress as growth-oriented adaptation. Organizationally, the disciplinary and evaluative structures surrounding AEs may have limited the expression or persistence of intermediate recovery positions. Rather than indicating the nonexistence of survival experiences, this pattern may reflect culturally and institutionally mediated ways of constructing and communicating recovery [22,26]. The observations in the study suggest that recovery trajectories following AEs are culturally mediated processes. Recognizing such influences helps situate the present findings within global Second Victim scholarship, highlighting that variations in recovery patterns may reflect differences in cultural and institutional contexts rather than mere deviations from universal models.

### Strengths and Limitations

The richness of the data ensured sufficiency, with participants working across six different provinces in China, offering a diverse cross-section in terms of geography, economic development, and healthcare infrastructure. However, several limitations should be acknowledged. First, the study focused on junior nurses involved in Grade III and IV AEs, which may limit the transferability of findings to more severe incidents. This predominance of mid-level events may also have contributed to the observed absence of the “survival” state in participants’ recovery trajectories, because such events typically involve less severe consequences and institutional scrutiny, reducing the likelihood that nurses experience the prolonged, intermediate coping stage characterized in Scott’s model. Future research examining recovery experiences following Grade I and II events would provide a more comprehensive understanding of second victim phenomena across differing levels of event severity. Second, the cross-sectional design precludes examination of how psychological responses and coping processes evolve over time. Longitudinal qualitative approaches would be valuable for capturing the dynamic nature of recovery trajectories. Finally, the interview timing relative to the occurrence of AEs was not standardized. Given the emotionally salient nature of AEs, participants’ accounts may have been influenced by recall bias, including memory attenuation, reconstruction, or retrospective reinterpretation. Additionally, some data collection occurred digitally with participants who did not activate their cameras, which may have introduced constrained expression. Prospective longitudinal research may further clarify how temporal distance from AEs shapes memory reconstruction, emotional responses, and recovery narratives.

## 5. Conclusions

Drawing on narratives from junior nurses and nurse managers across six provinces in China, this study demonstrates that early-career nurses may experience pronounced psychological distress even following predominantly minor AEs. Although participants’ accounts broadly resonated with Scott’s Second Victim Recovery Trajectory, recovery processes were primarily iterative and nonlinear. These findings underscore the significance of cultural and organizational contexts in shaping second victim experiences. Cultural norms, managerial practices, and institutional response patterns appear to influence how junior nurses interpret, cope with, and recover from AEs. The results imply the importance of timely and structured emotional support mechanisms, as well as the pivotal role of nurse managers in cultivating psychologically safe environments.

## Figures and Tables

**Table 1:** Semi-structured interview guide.

Questions Type	Questions
Initial Questions	Please talk about your current job.
	What has been the biggest challenge you’ve encountered since starting work?
Main Questions	How did the nursing adverse event occur?
	How did you feel after it happened? How did you get through this period?
	What was the hospital’s and department’s response? What actions were taken?
	How did your family and friends view this incident?
	What impact has this incident had on your future work?
Closing Questions	What advice would you give to newly recruited nurses? (What would you like to tell newly recruited nurses?)
	Are there any other important issues or experiences you think we haven’t discussed yet?

**Table 2:** Demographic characteristics of participants.

Participant	Age	Department	Years of Service	Marital Status	Current Job Title	Type of Employment	Monthly Income (CNY)	Type of Living Arrangement	Province of Work Location	Hospital Classification Level
L1	25	Dermatology Outpatient	2	Single	Nurse	Contractual	5000– 10,000	Living Alone	Unknown	Class 3B
P	26	Respiratory Medicine	1	Single	Nurse	Contractual	5000– 10,000	Living Alone	Guangdong	Class 3A
J	23	Respiratory Intensive Care Unit	1	Single	Nurse	Contractual	<5000	Living Alone	Shaanxi	Class 2A
W	23	Dermatology	1	Single	Nurse	Contractual	<5000	Living Alone	Guangdong	Class 3A
S	27	Urology	2	Single	Nurse	Contractual	5000– 10,000	Living with Others	Guangdong	Class 3A
L	23	Respiratory and Critical Care Medicine	1	Single	Nurse	Contractual	10,000– 15,000	Living Alone	Ningxia	Class 3A
F	21	General Surgery	1	Single	Nurse	Contractual	5000– 10,000	Living Alone	Gansu	Class 3A
X	25	Interventional Medicine	3	Single	Nurse	Contractual	5000– 10,000	Living Alone	Guangdong	Class 3A
D	25	General Practice	1	Married	Nurse	Full-time	<5000	Living with Family	Gansu	Class 2B
H1	34	Hepatobiliary Surgery	13	Married	Head Nurse	Full-time	>15,000	Living with Family	Guangdong	Class 3A
H2	55	Geriatrics	35	Married	Head Nurse	Full-time	>15,000	Living with Family	Jiangsu	Class 3A
H3	34	Thoracic Surgery	12	Married	Head Nurse	Full-time	>15,000	Living with Family	Guangdong	Class 3A
H4	50	Nursing Department	30	Married	Director Nurse	Full-time	>15,000	Living with Family	Sichuan	Class 3A

## Data Availability

The data presented in this study are available upon request from the corresponding author due to privacy and confidentiality constraints.

## Appendix A

**Table A1:** Grade of AEs and related penalties.

Grade	Definition	Penalties
IV	Preventable events attributed to environmental or systemic factors that do not harm the patient.	Verbal warning issued within the department.
III	Events causing no harm or only minor injury to the patient.	1. Verbal departmental warning. 2. Written self-reflection report. 3. Discretionary deduction from the individual’s bonus.
II	Events resulting in serious injury or posing life-threatening risk.	1. Hospital-wide public censure. 2. Suspension from clinical practice for 6–12 months or revocation of practice license. 3. Depending on responsibility: warning, demotion, removal from post, or dismissal. 4. Ineligibility for nursing awards. 5. Career restrictions: (a) barred from promotion or professional-title evaluation for 1–2 years; or (b) demoted or suspended from higher appointments for 1–3 years.
I	Events resulting in the patient’s death.	1. Hospital-wide public censure. 2. Suspension from clinical practice for 6–12 months or license revocation. 3. Depending on responsibility: warning, demotion, removal from post, or dismissal. 4. Demotion for 1–3 years. 5. Loss of professional qualifications for 1–2 years. 6. Civil compensation proportional to fault. 7. Criminal liability in severe cases.

## Appendix B. The Skeletal Narrative of the AE from Nurse W

Nurse W, a 23-year-old female nurse working in the dermatology department of a tertiary hospital, with a few years of experience, was involved in an adverse event where a patient fell. The incident occurred while Nurse W was in the process of handing over her shift after working a night shift. During the handover, an irate family member approached the nurse station and questioned Nurse W about why she hadn’t been aware of the patient’s fall. Upon hearing this, the head nurse, Nurse W, and several colleagues rushed to the patient’s ward to assess the situation. Nurse W recalled feeling both anxious and frightened at the moment, noting that a fall could range from minor to very serious. She explained that if the patient had fallen on their head, the consequences could have been severe, particularly given the patient’s critical condition and advanced age.

Fortunately, the patient had only injured their hip joint. Nurse W immediately called a doctor and arranged for a CT scan. An investigation into the cause of the fall revealed that the patient had tripped over a plastic bag that had been placed by the night shift nurse in the bedside drawer the previous day. The patient or the patient’s family had later moved the bag to the foot of the bed. When the patient got up to use the restroom the next morning, the plastic bag got caught on the patient’s foot, causing the fall. Nurse W had previously instructed both the patient and family members not to attempt activities alone, but still felt regretful about the incident. She reflected that the incident might not have occurred if she had conducted more frequent rounds and been more attentive during them.

After the incident, the head nurse privately reprimanded Nurse W, asking for clarification about the placement of the plastic bag. Nurse W reported feeling upset and “wronged,” describing how the head nurse pulled her aside after the handover and scolded her again while repeatedly questioning the placement of the bag. She also felt “unlucky,” noting that the incident occurred during her night shift when she was already exhausted. She added that she had to apologize to the patient’s family and seek their forgiveness.

Since the CT results for the patient had not been received at the time, Nurse W was anxious about potential complications. After leaving the hospital, she continued to call her colleagues for updates until one of them confirmed that everything was fine. Only then was she able to relax.

Following the incident, the department held a full team discussion and analysis of the event. Nurse W became much more conscientious about patient rounds in her future work, stating that this incident served as a wake-up call that made her more attentive and diligent in her duties moving forward.
